# Latent tuberculosis prevalence in healthcare workers in Laos: a cross-sectional study

**DOI:** 10.1186/s41182-024-00677-2

**Published:** 2025-01-03

**Authors:** Sypaseuth Duangmixay, Siriphone Virachith, Judith M. Hübschen, Phitsada Siphanthong, Sakhone Suthepmany, Somphou Sayasone, Antony P. Black

**Affiliations:** 1 Lao Tropical Public Health Institute, Vientiane, Laos; 2 LaoLuxLab/Vaccine Preventable Diseases Laboratory, Institut Pasteur du Laos, Vientiane, Laos; 3 Luxembourg Institute of Health, Esch-Sur-Alzette, Luxembourg; 4 National Tuberculosis Control Centre, Vientiane, Laos; 5 School of Life Sciences, Westminster University, London, UK

**Keywords:** Latent tuberculosis, Healthcare workers, Lao People’s Democratic Republic

## Abstract

**Background:**

Individuals with latent tuberculosis infection (LTBI) have a high risk of active infection, morbidity and mortality. Healthcare workers are a group who have increased risk of infection and onward transmission to their patients and other susceptible individuals; however, LTBI is often undiagnosed, and individuals are asymptomatic. Interferon gamma release assays (IGRA) can detect evidence of TB infection in otherwise asymptomatic individuals and are a good indication of LTBI. Laos, a resource limited country in southeast Asia, has limited data on TB prevalence in the general population or in healthcare workers. This study aimed to estimate the prevalence of LTBI in Lao healthcare workers in Vientiane Capital.

**Methods:**

Healthcare workers from high-risk departments from 3 central hospitals in Laos were included (n = 196) and venous blood was tested by IGRA. A questionnaire was administered to determine their knowledge, attitude and practice towards TB and LTBI.

**Results:**

10.2% of the participants were positive by IGRA, none of whom were previously aware of their TB status. The questionnaire revealed that knowledge and awareness of TB and LTBI were low.

**Discussion:**

A significant proportion of healthcare workers in this study had evidence of LTBI infection. These individuals were unaware of their TB status and we suggest that testing and treatment, as well as prevention strategies, should be routinely administered in Lao hospitals.

## Background

Tuberculosis (TB), caused by the *Mycobacterium tuberculosis* bacteria, is one of the leading causes of infectious disease mortality worldwide, with approximately 10.6 million cases and 1.3 million deaths in 2022 [1]. Latent TB infection (LTBI) is defined by the World Health Organization (WHO) as a state of persistent immune stimulation by *Mycobacterium tuberculosis*, without any clinical symptoms of active TB [2]. Approximately one quarter of the world’s population may have LTBI and around 10% of these will progress to develop active TB, which has a mortality rate of about 50% if untreated [2–4]. In line with the WHO End TB strategy, LTBI individuals should be diagnosed and treated appropriately, particularly in high-risk sub-populations, due to their risk of developing active TB and possibility of spreading to susceptible individuals [2]. Healthcare workers, especially those with close patient contact, are an important risk group for TB and LTBI and have been shown to be at higher risk of infection [5]. Furthermore, infected healthcare workers pose a risk of onward transmission to their patients if LTBI becomes reactivated.

Laos is a lower-middle-income country within the WHO Western Pacific Region and bordering the Southeast Asia region. Both these regions have estimated LTBI rates of over 20%, although this varies greatly by study [3]. Routine vaccination in Laos is challenging, with low vaccine coverage contributing to recent outbreaks of diphtheria [6], polio [7], measles [8] and pertussis [9]. Furthermore, Lao healthcare workers have been previously shown to be susceptible to vaccine-preventable infectious diseases, such as hepatitis B and diphtheria [10].

There are few data concerning the prevalence of TB in Laos and no published studies to date on the prevalence on LTBI in Lao healthcare workers. The aim of this study was to estimate the prevalence of LTBI in Lao healthcare workers.

## Methods

A cross-sectional study was done between March and June 2023. Healthcare workers were recruited from Mitthaphab, Mahosot and Setthathirath Central hospitals in Vientiane Capital. A convenience sampling approach was taken as follows. Departments for recruitment were selected according to whether they had healthcare workers in close contact with patients. All healthcare workers aged 18 years and over were invited, unless they had confirmed TB infection. Thus, a total of 196 healthcare workers were included from the infectious diseases, pulmonary, emergency, intensive care and out-patient departments. Following signed informed consent, 5 mL venous blood was taken. Blood was sent to the Institut Pasteur du Laos and the quantiferon-TB Gold Plus assay (Qiagen) was performed as per the manufacturer’s instructions. A short questionnaire was administered to determine the participants’ demographics, as well as knowledge, attitude and practice of the healthcare workers towards TB and LTBI. The questions included; history of close contact with TB patients or family; protective measures used within the hospital setting. Healthcare workers with LTBI were referred to the appropriate departments for follow-up and X-ray (Table 1).

**Table 1:** Participant demographics and characteristics, *N* = 196

Variable	N (%)
Sex	
Male	36 (18.4)
Female	160 (81.6)
Age group (years)	
18–40	126 (64.3)
41–60	69 (35.2)
Over 60	1 (0.5)
Ethnicity	
Lao-tai	185 (94.4)
Non-Lao-tai	11 (5.6)
Marital status	
Married	128 (65.3)
Unmarried	68 (34.7)
Education level	
Undergraduate	107 (54.6)
Bachelor	59 (30.1)
Master	30 (15.3)
Occupation	
Doctor	60 (30.6)
Nurse	136 (69.4)
Work duration	
< 5 years	65 (33.2)
5–10 years	45 (23.0)
11–20 years	47 (24.0)
Over 20 years	39 (19.9)
History of close contact with TB cases	
Household contact	6 (3.1)
BCG at birth (recall and scar)	149 (76.0)

## Results

In total, 20/196 (10.2%) of participants had positive IGRA. There were positive, though not significant associations with increasing age, being male, working for longer in health facilities and contact with TB infected individuals at work or home (data not shown).

Although the participants’ knowledge of TB, as assessed by questionnaire, was adequate, we did identify knowledge gaps which could be addressed with awareness campaigns and training for risk management. For example, 69.9% of the participants did not know the definition of multi-drug-resistant TB, 58.7% did not know the standard treatment for TB and 38.3% did not know the definition of LTBI (Table 2).

**Table 2:** Knowledge, attitude and practice of participants

Question	N (%)
Worried about getting TB	148 (75.5)
Would like to be regularly tested for TB	182 (92.9)
Feel that TB is a major threat to public health in Laos	183 (93.4)
Think that there is a need to improve TB control in region	193 (98.5)
Know the correct definition for LTBI	121 (61.7)
Know the standard treatment for TB	81 (41.3)
Know the definition of multi-drug-resistant TB	59 (30.1)

## Discussion

To our knowledge, this is the first study in Laos to determine the prevalence of LTBI in healthcare workers. In this study, we found that 10.2% of healthcare workers from central hospitals in Vientiane Capital, Laos, had IGRA positive results. None of these participants were aware of their TB status and none reported symptoms, indicating that these were LTBI cases. It is possible that a small proportion may have low level active TB and, indeed, approximately 5–15% of individuals with LTBI progress to active TB [5].

Other studies in neighboring countries have found a range of LTBI rates among healthcare workers such as 28.6% in China [12] and 14.7–40.1% in Thailand [13, 14]. These varying rates underline the different levels of TB exposure in different settings in healthcare workers and in the general public. Although the LTBI levels in our study are lower than reported in other countries in the region, they are still significant and cause for concern. Risk factors for LTBI in healthcare workers in other countries included age, duration of employment [13], performing aerosol generating procedures whilst in contact with TB cases and absence of BCG scars [15].

One previous study in Laos in 2009 in Lao children living in households with TB patients found low levels of awareness of TB infectiousness among patients and their contacts and frequent risky behavior [11]. Our data also show that the knowledge of Lao healthcare workers is relatively low, with many unaware of the definition of LTBI and unsure regarding TB treatment. A low knowledge of treatment was also found in neighboring Cambodia, with suggestions for training of caregivers and increased awareness of risk practices [16].

The main limitation of this study is the small sample size. As such, we could not do risk factor analysis and caution should be applied when extrapolating the data to other settings within the country. The research needs to be expanded in Laos on a larger scale and include healthcare settings other than the Central Hospitals.

In Laos, there is no current policy to screen for LTBI among healthcare workers, mainly due to limited resources. Early diagnosis and proper management of TB and LTBI are crucial for its control in healthcare and other settings. Our preliminary data underline the need for routine screening of healthcare workers in Laos for LTBI. Nevertheless, the scaleup of LTBI activities for both healthcare workers and patients have significant cost and human resource implications that need to be considered [17]. Risk management strategies, including appropriate personal protective equipment, should be enforced in addition to proper case management and awareness campaigns.

## Data Availability

The anonymized data sets used and analysed during the current study are available from the corresponding author on reasonable request.

## Abbreviations

LTBI: Latent tuberculosis infection
TB: Tuberculosis
WHO: World Health Organization
